# STAT1 deficiency underlies a proinflammatory imprint of naive CD4^+^ T cells in spondyloarthritis

**DOI:** 10.3389/fimmu.2023.1227281

**Published:** 2023-10-18

**Authors:** Bilade Cherqaoui, Frédéric Crémazy, Marc Lauraine, Ghazal Shammas, Roula Said-Nahal, Hendrick Mambu Mambueni, Félicie Costantino, Marine Fourmont, Audrey Hulot, Henri-Jean Garchon, Simon Glatigny, Luiza M. Araujo, Maxime Breban

**Affiliations:** ^1^ Infection & Inflammation, UMR 1173, Inserm, UVSQ/Université Paris Saclay, Montigny-le-Bretonneux, France; ^2^ Laboratoire d’Excellence Inflamex, Université Paris-Centre, Paris, France; ^3^ Rheumatology Division, Ambroise Paré Hospital, AP-HP, Boulogne-Billancourt, France; ^4^ Genomic Platform of Faculty of Health Simone Veil, UVSQ/Université Paris Saclay, Montigny-le-Bretonneux, France

**Keywords:** ankylosing spondylitis, dendritic cells, epigenomic, HLA-B27, naive CD4 + T cells, STAT1, spondyloarthritis, T helper 17

## Abstract

**Introduction:**

In spondyloarthritis (SpA), an increased type 3 immune response, including T helper cells (Th) 17 excess, is observed in both human and SpA animal models, such as the HLA-B27/human β2-microglobulin transgenic rat (B27-rat).

**Methods:**

To investigate this unexplained Th17-biased differentiation, we focused on understanding the immunobiology of B27-rat naive CD4^+^ T cells (Tn).

**Results:**

We observed that neutrally stimulated B27-rat Tn developed heightened Th17 profile even before disease onset, suggesting an intrinsic proinflammatory predisposition. In parallel with this observation, transcriptomic and epigenomic analyses showed that B27-rat Tn exhibited a decreased expression of Interferon/Th1- and increased expression of Th17-related genes. This molecular signature was predicted to be related to an imbalance of STAT1/STAT3 transcription factors activity. *Stat1* mRNA and STAT1 protein expression were decreased before disease onset in Tn, even in their thymic precursors, whereas *Stat3*/STAT3 expression increased upon disease establishment. Confirming the relevance of these results, *STAT1* mRNA expression was also decreased in Tn from SpA patients, as compared with healthy controls and rheumatoid arthritis patients. Finally, stimulation of B27-rat Tn with a selective STAT1 activator abolished this preferential IL-17A expression, suggesting that STAT1-altered activity in B27-rats allows Th17 differentiation.

**Discussion:**

Altogether, B27-rat Tn harbor a STAT1 deficiency preceding disease onset, which may occur during their thymic differentiation, secondarily associated with a persistent Th17 bias, which is imprinted at the epigenomic level. This early molecular phenomenon might lead to the persistent proinflammatory skew of CD4^+^ T cells in SpA patients, thus offering new clues to better understand and treat SpA.

## Introduction

Spondyloarthritis (SpA) is a frequent chronic inflammatory rheumatism that primarily affects the axial skeleton and, frequently, the peripheral joints and extra-articular tissues, including psoriasis, anterior uveitis, and inflammatory bowel disease (IBD) (1). Its striking association with the human leukocyte antigen (HLA)–B27 remains incompletely understood (2).

Several lines of rats transgenic for HLA-B27 and human β2-microglobulin (hβ2m) (B27-rat) spontaneously develop inflammatory manifestations recapitulating the main SpA features (rat-SpA) in a specific way since HLA-B7/hβ2m-transgenic rats (B7-rats) remain healthy (3). Thus, B27-rat represents a faithful model to study the pathogenicity of HLA-B27 in SpA (4). Contrary to expectation for a class I major histocompatibility complex-associated disease, previous studies evidenced extensively that CD8^+^ T cells were dispensable, but that CD4^+^ T cells were critical in mediating disease manifestations (3). Further characterization of effector CD4^+^ T cells showed a selective expansion of proinflammatory CD4^+^ T cells exhibiting a T helper 17 (Th17) phenotype (5).

Furthermore, antigen-presenting cells (APCs), including dendritic cells (DCs), from disease-prone B27-rats were shown to drive CD4^+^ T-cell polarization toward Th17 bias, supporting the hypothesis that the expression of HLA-B27 in APC plays a critical role in triggering the inflammatory process (5, 6). Moreover, several defective functions of DCs have been identified in the B27-rat, which may promote skewed T-cell differentiation (7, 8).

In the present study, our objective was to decipher the molecular mechanisms favoring an altered differentiation of naive CD4^+^ T cells (Tn) from B27-rat. We first studied the influence of DCs on Tn fate, then focused on intrinsic predisposition of Tn to differentiate into Th17 cells, using transcriptomic, epigenomic, and functional approaches. This allowed us to identify decreased expression of STAT1 in Tn from B27-rat as an early molecular event underlying Th17 differentiation bias. We finally identified that decreased *STAT1* gene expression was also present in Tn from SpA patients, strengthening the relevance of our finding for SpA.

## Materials and methods

### Rats

The SpA-prone B27-rats of the 33-3 line bearing 55 copies of HLA-B*2705 and 28 copies of hβ2m and the healthy B7-rats of the 120-4 line bearing 52 copies of HLA-B*0702 and 26 copies of hβ2m, all on a Fisher (F344) background, were bred under conventional conditions (5). Age-matched nontransgenic (NTG) littermates were used as controls. B27-rats were used at three distinct ages: (i) ≤ 10 days, asymptomatic, so-called newborn; (ii) 3–4 weeks, asymptomatic, so-called premorbid; and (iii) 2–12 months, sick. Study procedures were approved by the Institutional Animal Experimentation Ethical Committee from the Faculty of Health Simone Veil (APAFIS-8910).

### Rat cells preparation

Single-cell suspensions were prepared mechanically either from mesenteric lymph nodes (mLN), cervical LN (cLN), or thymus. Subsets of CD4^+^ T cells were purified by flow cytometry (ARIA-III). TCRαβ^+^CD4^+^CD25^−^CD62L^high^ mature Tn were isolated from LNs (**Supplementary Figure S1A**). The sorted T cells were uniformly CD44^low^, confirming that they corresponded to Tn, rather than central memory T cells (data not shown). TCRαβ^+^CD4^+^CD8^−^ and TCRαβ^+^CD4^+^CD8^+^ thymocytes were isolated from the thymus. Conventional type-2 DCs (cDC2) were obtained from the spleen, digested with collagenase-D (2 mg/mL, Roche, Mannheim - Germany) (9). Low-density cells were collected after centrifugation on a 14.5% Nycodenz gradient (Nycomed, Oslo - Norway), magnetically enriched for CD103^+^ subset (anti-rat microbeads, Miltenyi—autoMACS Pro Separator, Bergisch Gladbach - Germany) and sorted by flow cytometry as TCRαβ^−^CD45RA^−^CD4^+^CD103^+^ cells. Fluorochrome-conjugated mouse anti-rat TCRαβ, CD103 were from BioLegend; mouse anti-rat CD4, CD45RA from BD (San Jose - California/US); mouse anti-rat CD62L, CD25, mouse anti-rat CD8 from Thermo Fisher Scientific (Eugene - Oregon/US).

### Culture of rat Tn

Tn were labeled with fluorochrome-conjugated CellTraceViolet (CTV, Thermo Fisher Scientific) according to the manufacturer’s instructions, then cultured (10^5^ per well) with soluble anti-rat CD28 (1 µg/mL, BD) in round-bottom 96 wells plate coated with mouse anti-rat CD3 (2 µg/mL, BD), in complete medium was RPMI-1640 with Glutamax I (Life Technologies, Paisley - United Kingdom), 10% fetal calf serum, streptomycin/penicillin (100 µg/mL), 2% sodium pyruvate, 0.05 mM β-mercaptoethanol and 10 mM HEPES (complete RPMI), during 3 or 6 days. In some experiments, rat recombinant (rr) IL-1β (20 ng/mL, R&D Systems, Minneapolis - Minnesota/US), rrIFN-γ (10 ng/mL, Miltenyi), STAT1 transcriptional activator, 2-(1,8-Naphthyridin-2-ly)phenol [2NP] (50µM, Abcam, Cambridge - United Kingdom) were added at the beginning of culture (10, 11). 2NP concentration was optimized in preliminary experiment, confirming a significant increase of pSTAT1 expression level in Tn, from 51.1% to 68.7% at 50 µM dose (**Supplementary Figure S1B**). Th1 differentiation was induced by adding rrIL-12 (10 ng/mL, R&D Systems), Ab neutralizing rat IL-4 (1 µg/ml, Miltenyi), and rrIL-2 (5 ng/ml, ImmunoTools, Friesoythe - Germany) at the beginning of a 4-day culture.

### Coculture of rat Tn and cDC2

After overnight maturation of cDC2 (37°C) with rr granulocyte macrophage colony stimulating factor (100 ng/mL, BioLegend, San Diego - California/US), 10^5^ mLN Tn labeled with CTV were added to 10^4^ cDC2 in complete RPMI, in the presence of soluble anti-TCRαβ (1µg/mL, BioLegend), during 3 or 6 days.

### Rat T-cell immunophenotyping

After culture, T cells were stimulated with phorbol myristate acetate (10 ng/mL, Sigma-Aldrich, Saint-Louis - Missouri/US), ionomycin (1 µg/mL, Sigma-Aldrich) for 4h, in the presence of brefeldin A (2 µg/mL, Sigma-Aldrich). Live-Dead and CTV staining identified live cells that had divided. Cell surface activation markers (CD25, ICOS)—fixed before permeabilization (buffers from Tonbo, San Diego - California/US)—or intra-cellular cytokines (IL-17A, TNFα, and IFNγ) expression was evaluated by flow cytometry (Fortessa). We then analyzed T-cell immunophenotyping using FlowJo (version 10, BD), gating on live CD4^+^ T cells. Isotypes were used to define gating strategy for CD25, ICOS, IL-17A, and TNFα staining (**Supplementary Figure S1C**). Mouse anti-rat IFN-γ was from BD; mouse anti-rat ICOS, IL-17A, and hamster anti-rat TNFα, IL-17A enzyme-linked immunosorbent assay (ELISA) kit from Thermo Fisher Scientific.

### Human T-cell preparation

Axial SpA patients -fulfilling the Assessment of SpondyloArthritis International Society classification criteria (12)-, age/sex-matched healthy controls (HCs) and rheumatoid arthritis (RA) patients -fulfilling the American College of Rheumatology/European League Against Rheumatism classification criteria (13)- were recruited in the department of rheumatology of Ambroise Pare hospital. Clinical data are summarized in **Supplementary Table S1**. Peripheral venous blood peripheral blood mononuclear cells (PBMCs) were isolated by Ficoll (GE Healthcare, Chicago - Illinois/US) density gradient centrifugation. CD14^+^ cells were removed by positive magnetic selection (AutoMacs) using human anti-human CD14 microbeads (Miltenyi). Total CD14^−^ PBMC were used for *ex-vivo* (p)STAT1/3 assay. CD4^+^CD45RA^high^CD45RO^low^CCR7^+^ Tn were sorted by flow cytometry (**Supplementary Figure S1D**) for quantitative real-time polymerase chain reaction (q-RT-PCR). Anti-human CD4, CD45RA, CD45RO, and mouse anti-human CCR7 were from BD. Written informed consent was obtained before study, approved by the local ethical committee (Comité de Protection des Personnes Ile-de-France XI, AOR10006-NI09031).

### Total STAT1 and STAT3 intra-cellular content

Single-cell suspensions of rat mLN were marked to identify CD4^+^CD25^−^CD62L^+^ Tn. After fixation, permeabilization (buffers from BD) and staining with anti-total STAT1 and STAT3 Abs (BD), the mean fluorescent intensity (MFI) was quantified by flow cytometry (Fortessa).

### STAT1 and STAT3 phosphorylation

Single-cell suspensions of rat mLN were stained to identify CD4^+^CD25^−^CD62L^+^ Tn. Then, they were stimulated with mouse rIL-27 (5 ng/mL, R&D Systems) or rrIL-6 (10 ng/mL, R&D Systems) for 10 min at 37°C. After fixation, permeabilization (buffers from BD) and staining with mouse anti-human/mouse STAT1 phosphorylated at tyrosine 701 and STAT3 phosphorylated at tyrosine 705 Abs (BD), the MFI was quantified by flow cytometry (Fortessa).

### RNA sequencing

Total RNA was purified from mLN Tn sorted from 4-week- (Monarch Total RNA Miniprep Kit, NEB, Ipswich, Massachusetts/US) or 3-month-old rats (RNeasy Micro Kit, QIAGEN, Hilden - Germany). Samples with RNA integrity number > 7 were sequenced. Libraries were prepared using NEBNext Low Input RNA Library Prep Kit (3-month-old) or NEBNext rRNA Depletion Kit and NEBNext Ultra II Directional RN Library Prep Kit (4-week-old), and sequenced on an Illumina HiSeq 2000 (3-month-old) or NextSeq 500/550 (4-week-old), paired-end 75 bases. Sequencing was performed at the genomic platforms of Cochin Institute—Paris Centre University, or Faculty of Health Simone Veil–Versailles-Saint-Quentin/Paris-Saclay University. Volcano plots were designed with EnhancedVolcano. Transcripts were aligned to the rat genome Rnr_6.0 using STAR version 2.7.1 then reads were counted using featureCount (14, 15). Differential analysis was performed with DESeq2 including batch effect (16). Pathways analysis was performed using QIAGEN Ingenuity Pathway Analysis (IPA) (17, 18). Upstream transcription regulators were retrieved using IPA, computing an overlap *p*-value (overlap between the dataset genes and the genes regulated by a transcription regulator) and an activation *z*-score, which infers the predicted activation state of the transcription regulator.

### q-RT-PCR

Tn and thymocyte pellets were collected in TRIzol RNA Isolation Reagents (Thermo Fisher Scientific), then RNA was extracted using chloroform/ethanol (rat) or Monarch Total RNA Miniprep Kit (human). cDNA was synthesized using RevertAid First Strand cDNA Synthesis Kit (Thermo Fisher Scientific), then q-RT-PCR was performed using SsoAdvanced™ Universal SYBR Green Supermix (BioRad, Hercules - California/US) and CFX384 (Touch Real-Time PCR Detection System). Duplicates were run for each sample; *Gapdh*/*GADPH* and *Actin*/*ACTIN* were used as endogenous reference genes for mRNA abundance normalization (8). Primer sequences are provided in **Supplementary Table S2**.

### Chromatin immunoprecipitation followed by whole-genome sequencing

mLN Tn (2–7 × 10^5^ cells) were sorted from 4-week- or 3-month-old rats, fixed using 1% paraformaldehyde (Electron Microscopy Sciences, Dresden - Germany) for 8 min. After cell nuclei lysis, chromatin was fragmented (Bioruptor PICO/Diagenode, Seraing - Belgium), immunoprecipitated overnight at 4°C using rabbit polyclonal Abs: IgG (Diagenode, 1 µg), m/r anti-H3K4Me3 (Diagenode, 1 µg), m/r anti-H3K27Ac Abs (Active Motif, 5 µg, Carlsbad California/US), then tagmented using the ChIP-mentation Kit for Histones (Diagenode). Libraries were prepared according to the ChIP-mentation Kit guidelines and sequenced using the NextSeq 500 (Illumina). Sequencing was performed at the genomic platforms of Integrative Institute of Cellular Biology – Paris-Saclay University, or Faculty of Health Simone Veil–Versailles-Saint-Quentin/Paris-Saclay University. Reads were trimmed with fastp and aligned to the rat genome Rnr_6.0 using Bowtie2 (19, 20). The peak calling was performed with MACS2, normalized on IgG signal (21). Peaks were visualized using IGV software (22). Distribution across the different genomic features was performed using the R package ChIPseeker (23). Differential peaks enrichment analysis was performed using DiffBind (24). Superenhancers (SEs) were retrieved using ROSE, after enhancers ranking depending on H3K27Ac signal (25). Transcription factors binding motif were scanned using Hypergeometric Optimization of Motif EnRichment (HOMER) (26, 27). Gene Ontology (GO) and Kyoto Encyclopedia of Genes and Genome (KEGG) pathways analysis was performed using g: Profiler. Upstream transcription regulators were retrieved using IPA, as previously described (18).

### Statistical analysis

Flow cytometry and q-RT-PCR data were analyzed using GraphPad-Prism 9. Significance was determined by paired or unpaired Student’s t-test; Wilcoxon test; one- or two-way analysis of variance followed by Tukey’s multiple comparisons test; simple linear regression. *p*-values were adjusted with Benjamini–Hochberg process for RNA/ChIP-seq data. *p*-values were considered significant if < 0.05.

## Results

### B27-rat DCs and Tn jointly contribute to CD4^+^ T-cell proinflammatory skew

**Figure 1:**
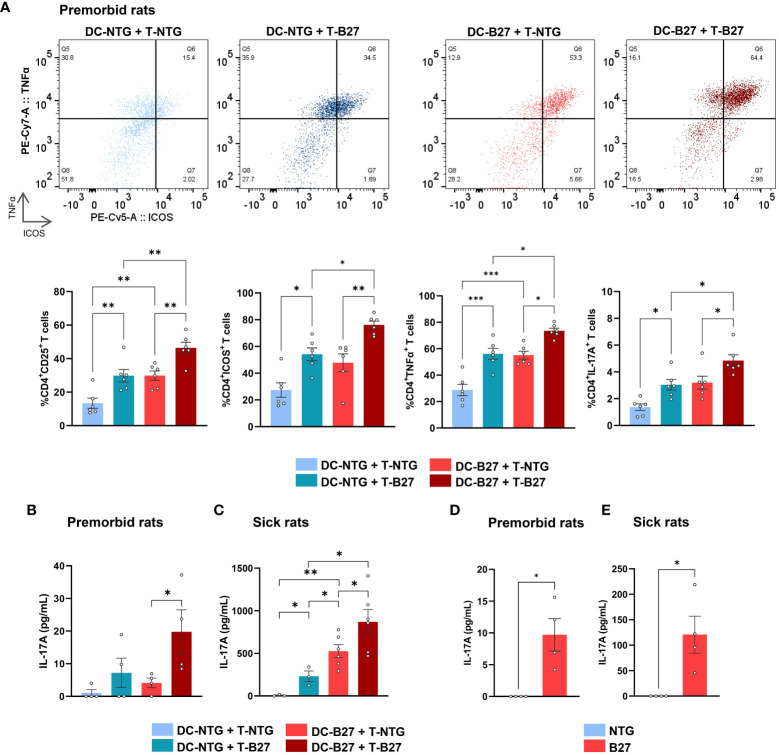
B27-rat DCs and Tn jointly contribute to T-cell proinflammatory skew. **(A–C)** Cocultures of splenic cDC2 with mLN Tn or **(D, E)** monoculture of stimulated mLN Tn were performed. Cells were harvested from **(A, B, D)** premorbid or **(C, E)** sick B27-rats and age-matched NTG littermates (*n* = 4–6). **(A)** After 3 days, cell surface or intra-cellular expression of CD25, ICOS, TNFα, and IL-17A was analyzed by flow cytometry, gated on live CD4^+^ T. Upper panel shows a representative dot plot of ICOS/TNFα expression in live CD4^+^ T cells; lower panel displays bar charts synthesis. **(B–E)** After 6 days, IL-17A was assessed by ELISA in supernatants. Bar charts represent the mean ± SEM; each dot represents an individual animal. **(A–C)** Two-way analysis of variance showed significant effect of DC genotype (*p* ≤ 0.0001) and T-cell genotype (*p* ≤ 0.001) in all comparisons, without significant interaction for **(A)** CD25, ICOS, TNFα and IL-17A expression (*p* = 0.2, 0.6, 0.7, and 0.2, respectively) or for **(B, C)** IL-17A production in premorbid and sick rats (*p* = 0.3 and 0.2, respectively). Groups comparisons were performed by **(A–C)** Tukey’s test or **(D, E)** unpaired t-test; *p < 0.05, **p < 0.01, and ***p < 0.001.

To examine the respective influence of Tn and DCs in the previously described Th17 differentiation bias (5), we performed cross-cultures of Tn with cDC2 from B27-rats or NTG littermates and analyzed CD4^+^ T-cell phenotype at the end of culture (**Figure 1A**). Both cDC2 and Tn from B27-rats contributed additively to the proinflammatory Th17-like profile of CD4^+^ T cells, characterized by heightened expression of activation markers such as CD25 and ICOS—the latest known to be critical for Th17 differentiation (28)—and inflammatory cytokines such as TNFα and IL-17A, even before disease onset. This was also confirmed by an increased production of IL-17A in culture supernatant (**Figure 1B**). Absolute numbers of Tn and effector CD4^+^ T cells were similar in the mLN of premorbid B27-rats and NTG littermates, showing that they had not undergone *in-vivo* pre-activation (**Supplementary Figure S2A**). Even stronger Th17-like profile of CD4^+^ T cells was observed after cocultures of cDC2 and Tn from sick B27-rats, showing that this phenomenon was amplified upon disease establishment (**Figure 1C**, **Supplementary Figure S2B**).

### B27-rat Tn are prone to Th17 differentiation

We next examined B27-rat Tn responses after *in vitro* activation in the absence of cDC2. Consistent with the foregoing results, stimulated Tn from both premorbid (**Figure 1D**, **Supplementary Figure S2C**) and sick B27-rats (**Figure 1E**, **Supplementary Figure S2D**) overexpressed activation markers and harbored a Th17-like phenotype. In contrast, Tn from healthy transgenic B7-rats behaved like NTG rats T cells (**Supplementary Figure S2E**).

**Figure 2:**
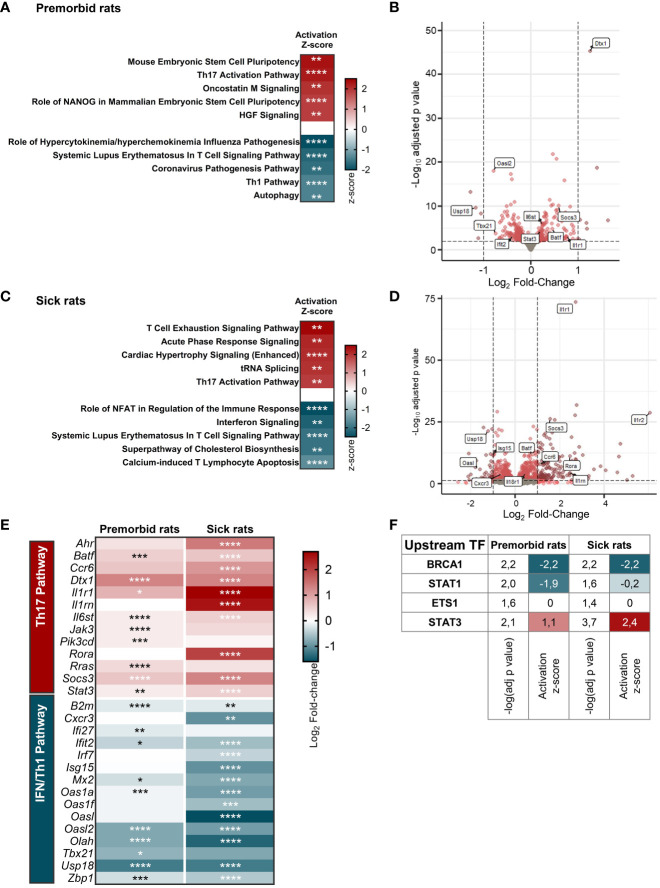
B27-rat Tn are prone to Th17 differentiation. RNA-seq was performed on mLN Tn from **(A, B, E, F)** 3-week-old premorbid B27-rats (*n* = 7) and NTG controls (*n* = 5), and **(C–F)** 3-month-old sick B27-rats (*n* = 7) and NTG controls (*n* = 7). **(A, C)** Top 5 IPA terms predicted to be the most activated and top 5 IPA terms predicted to be the most inhibited in B27-rats DEG, as compared to controls. The z-score refers to the activation (red) or inhibition (blue) state of the pathway in B27-rats compared to controls. *p < 0.05, **p < 0.01, ***p < 0.001, and ****p < 0.0001. **(B, D)** Volcano plot representing gene expression in Tn from B27-rats, as compared to controls. DEG involved in IFN/Th1 and Th17 pathways are labeled in boxes. **(E)** Expression heatmap showing the log_2_ fold-change expression of IFN/Th1 and Th17-pathways related genes in Tn from B27-rats before (premorbid) of after (sick) disease onset, as compared to controls. *p < 0.05, **p < 0.01, ***p < 0.001, and ****p < 0.0001. **(F)** Upstream transcription factors predicted to explain DEG profile in Tn from both premorbid and sick B27-rats, as compared to controls; −log(adj *p*-value) calculated by Fisher’s exact test; z-score refers to the activation (red), inhibition (blue) or undefined (white) state of the downstream regulated genes in B27-rats.

To determine the factors driving the propensity of B27-rat Tn to develop a proinflammatory phenotype upon stimulation, we compared the whole-transcriptome of mLN Tn between premorbid B27-rats and NTG littermates. Principal components analysis highlighted a clear-cut difference between B27-rats and NTG Tn (**Supplementary Figure S3**/upper panel). Ninety-nine genes were upregulated and 128 were downregulated in B27-rats (**Supplementary Table S3**). Among the pathways significantly enriched in differentially expressed genes (DEGs), we extracted the top 5 predicted to be activated and the top 5 predicted to be inhibited. Corroborating our previous results *in vitro*, the Th17 pathway was one of the most activated in B27-rats, whereas IFN/Th1 pathway (including anti-viral responses) was the most inhibited (**Figures 2A, B**, **Supplementary Table S4**), consistent with the fact that both lineages counter-regulate each other (29). In Tn from sick B27-rats, we observed a similar profile of Th17 pathway activation and IFN/Th1 pathway inhibition compared to NTG littermates (**Figures 2C, D**, **Supplementary Figure S3**/lower panel, and **Supplementary Table S5**). By mapping the top DEG related to Th17 and IFN/Th1 pathways, we confirmed such signature before disease onset, which intensified upon disease establishment (**Figure 2E**). Four upstream transcription factors were predicted to account for this transcriptomic signature in both premorbid and sick rats (**Figure 2F**). In B27-rat Tn, STAT1 was predicted to be less active and STAT3 more active, consistently with these cross-regulated transcription factors being critical for Th1 and Th17 differentiation, respectively (**Figure 2F**, **Supplementary Table S6**) (30, 31).

### Th17 imprint of B27-rat Tn is epigenetically determined

**Figure 3:**
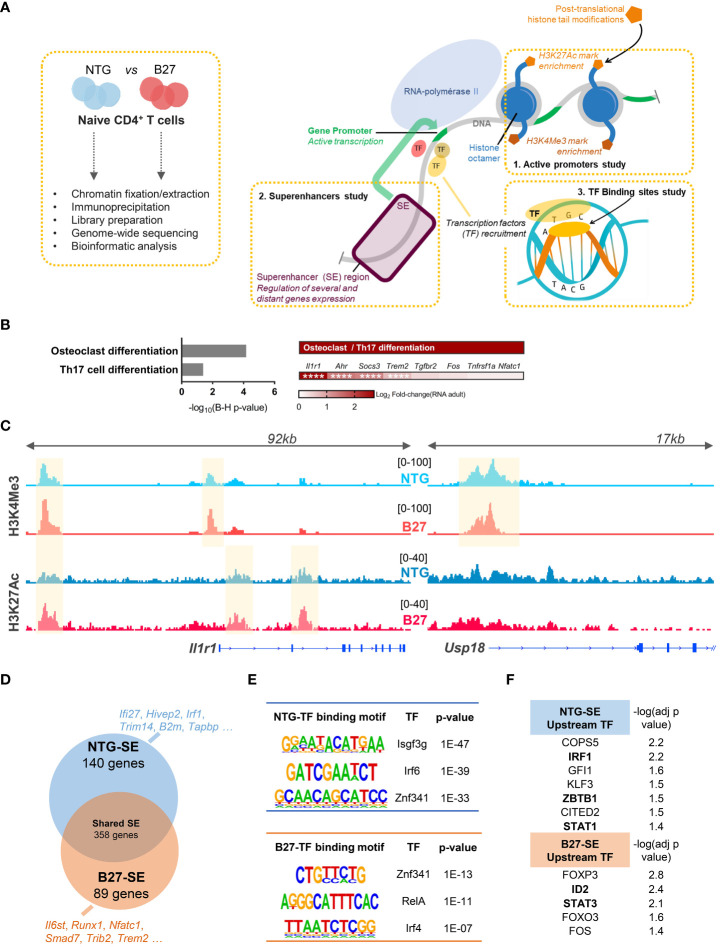
Th17 imprint of B27-rat Tn is epigenetically determined. H3K4Me3 and H3K27Ac activating HM ChIP-seq on mLN Tn from 3-month-old sick B27-rats and age-matched NTG littermates (*n* = 3). **(A)** Cartoon recapitulating the steps of ChIP-seq experiments and the features profiled: active promoters, superenhancers regions and binding transcription factors. (**B**/Left) KEGG pathways enriched for genes harboring a differential peaks enrichment of H3K4Me3 and/or H3K27Ac histone marks, in B27-rats compared to controls. (**B**/Right) Both KEGG pathways included the notified genes, and Log_2_FC(RNA) refers to the relative gene expression in Tn from B27-rats, as compared to controls from RNA-seq dataset. **** adjusted *p* < 0.0001. **(C)** Integrated genome viewer snapshots of genes involved in Th17 (*Il1r1*) and IFN (*Usp18*) pathways. Differentially enriched peaks at gene promoter (H3K4Me3) and/or presumed gene-related enhancer (H3K27Ac) are highlighted in yellow. Dataset from one pair of B27/NTG rats, representative of three. **(D)** Vein diagram showing the genes at proximity of the SE specific of B27-rat Tn and those specific of NTG littermates (using ROSE algorithm). Examples of SE-related genes specific of NTG and B27-rat Tn, involved in IFN (blue) or Th17 (red) pathways. **(E)** Examples of *de-novo* binding motifs of transcription factors predicted to fix on either NTG or B27-SE (using HOMER). **(F)** Upstream transcription factors predicted to regulate the expression of NTG or B27-rats SE-related genes (using IPA). −log (adj *p*-value) calculated by Fisher’s exact test. Transcription factors in bold if the transcript relative expression (foregoing RNA-seq data) was either decreased (NTG SE) or increased (B27-SE) in premorbid and sick B27-rat T cells relative to controls.

We next asked whether this transcriptomic signature of B27-rat Tn was reflected in their chromatin structure. We performed ChIP-seq to profile the enrichment of two activating histone marks in Tn from B27-rats and NTG littermates: H3K4Me3 and H3K27Ac, which are preferentially located on promoters and enhancers, respectively, to profile the epigenomic factors that might be involved in B27-rat Tn skewed differentiation (**Figure 3A**). Histone marks distribution across the different genomic features was comparable overall between B27-rats and NTG littermates (data not shown). The foregoing RNA-seq data evidenced that differential enrichment profile of histone marks between B27-rats and NTG littermates was not due to an alteration of histone modifiers expression (data not shown).

H3K4Me3 and H3K27Ac were differentially more enriched in Tn from sick B27-rats than in NTG in 26 and 126 sites, respectively (**Supplementary Figure S4A**, **Supplementary Table S7**). We next identified genes at proximity to these promoters and enhancers and pooled them to increase the power of KEGG pathways analysis. These genes were related to Th17 differentiation (**Figure 3B**), including *Il1r1* (**Figure 3C**, **Supplementary Figure S4C**), *Socs3*, *Ahr*, and *Trem2*, which were also upregulated at the transcriptomic level in B27-rat (**Figures 2D, E**). In NTG rat Tn, H3K4Me3 was more enriched only in six sites, including the *Usp18* (**Figure 3C**, **Supplementary Figure S4C**), *Olah* and *Oasl* genes, all related to the IFN pathway, which were also downregulated at the transcriptomic level in B27-rat (**Figures 2D, E**). B27-rat Tn harbored a biased epigenome including increased enrichment for H3K4Me3 histone marks at the *Il1r1* promoter, even before disease onset (**Supplementary Figure S4B**). Moreover, at the epigenomic level, Tn of healthy B7-rats, behaved like NTG rather than B27-rat Tn (**Supplementary Figure S4C**).

It is widely recognized that some enhancers can cluster to form large regulatory elements that facilitate the binding of numerous transcription factors and coactivators, leading to high expression level of genes, especially those involved in cell identity. These so-called SEs also play a key role during Tn differentiation (**Figure 3A**) (32). Indeed, differential SE activity could account for skewed T cell differentiation during SpA (33). We retrieved the SE based on H3K27ac enrichment and identified the closest potential target genes (**Supplementary Figure S4D**). RNA-seq data confirmed the higher transcriptional activity of genes near SE, compared to genes near conventional enhancers (**Supplementary Figure S4E**). We identified SE-related genes specific for either NTG or B27-rat Tn (**Figure 3C**, **Supplementary Figure S4D**, **Supplementary Table S8**). GO enrichment analysis showed that those B27-rat SE-related genes were involved in leukocyte differentiation and T-cell activation (data not shown), with several of them related to Th17 and NFκB pathways (**Figure 3D**). In contrast, several NTG rat SE-related genes were related to IFN pathway (**Figure 3D**). Next, we looked for *de novo* binding motifs of transcription factors that could preferentially bind on these SE (**Figure 3A**, **Supplementary Table S9**). Transcription factors critical for IFN pathway were predicted to bind to NTG SE, whereas transcription factors critical for Th17 and NFκB pathways were predicted to bind to B27-SE (**Figure 3E**). Among them, ZNF341, a regulator of STAT1 and STAT3 transcription activity (34), was predicted to bind to both NTG and B27-SE on distinct binding sites. Moreover, STAT1 and STAT3 were, respectively, predicted to be the upstream transcription factors regulating the expression of NTG or B27-rat SE-related genes (**Figure 3F**).

Even before disease onset, B27-rat Tn harbored specific activated SE related to genes with critical role for T cell activation and Th17 differentiation, such as *Ccr7*, *Il2ra*, *Tnfrsf14*, and *Stat3* (**Supplementary Table S10**). The *de novo* binding motifs of transcription factors that were predicted to preferentially bind to these B27-SE included IRF4, SMAD3, SMAD4, and BATF (**Supplementary Table S11**), which are known to be involved in Th17 differentiation and IBD (**Supplementary Figure S4F**).

Altogether, these data revealed that B27-rat Tn were imprinted at the epigenomic level, prior to disease onset, predisposing them to differentiate preferentially into Th17 cells. This could involve an imbalance of STAT1/STAT3 transcription factors activity, in favor of the latest.

### STAT1 deficiency is an early hallmark of B27-rat Tn

**Figure 4:**
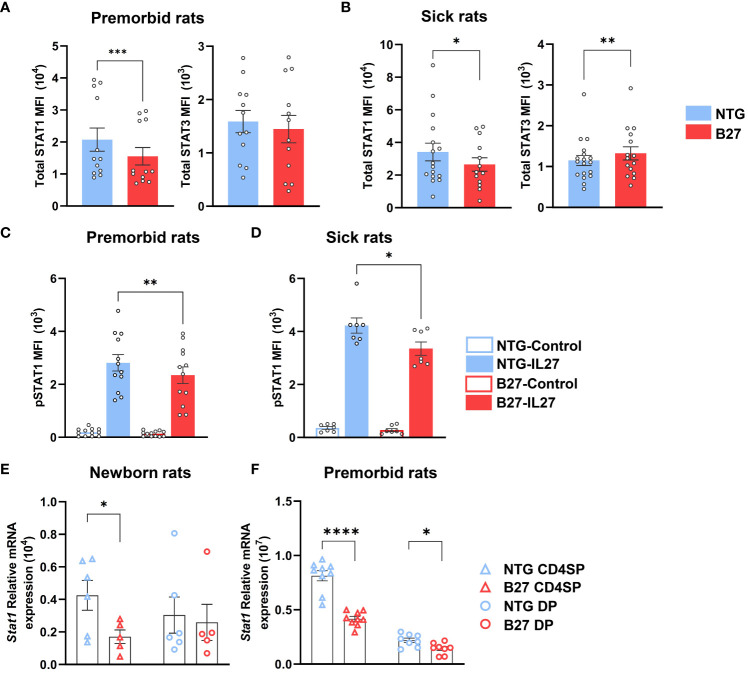
STAT1 deficiency is an early hallmark of Tn in B27-rat. **(A–D)** Single-cell suspension from mLN of **(A, C)** premorbid or **(B, D)** sick B27-rats, and NTG littermates. **(A, B)** Total STAT1 and STAT3 protein mean fluorescent intensity (MFI) assessed by flow cytometry in Tn. **(C, D)** Phosphorylated STAT1 MFI assessed by flow cytometry in Tn, after IL-27 stimulation for 10 min. **(E, F)** *Stat1* gene mRNA expression assessed by q-RT-PCR in CD4^+^ thymocytes (CD4SP) and CD4^+^CD8^+^ double-positive thymocytes (DP) of **(E)** newborn or **(F)** premorbid B27-rats and controls. Bar charts represent the mean ± SEM; each dot represents an individual animal. Statistical analyses were performed by using **(A)** Wilcoxon test or **(C, D)** paired *t*-test or **(E, F)** unpaired t-test; *p < 0.05, **p < 0.01, ***p <0.001 and ****p < 0.0001.

We further examined STAT1/STAT3 protein expression by flow cytometry in mLN Tn (**Supplementary Figures S5A, S5B**). Prior to disease onset, total STAT1 levels were decreased in B27-rat Tn, as compared to NTG littermates, whereas STAT3 levels were comparable between both rats Tn (**Figure 4A**). Following disease onset, the decrease of STAT1 expression persisted, while level of STAT3 became higher in B27- than in NTG rat Tn (**Figure 4B**). Confirming the specificity of these results, the expression levels of STAT5 and STAT6, critical for Treg and Th2 differentiation, respectively, were comparable between both rats Tn (**Supplementary Figure S5C**). As STATs are activated through phosphorylation, we examined the expression levels of pSTAT1 in Tn with or without exposure to IL-27, known to induce STAT1 phosphorylation and down-regulate Th17 differentiation in B27-rat model (**Supplementary Figures S6A–S6D**) (35, 36). STAT1 phosphorylation was lower after IL-27 exposure in B27-rat as compared to NTG Tn (**Figures 4C, D**). Conversely, pSTAT3 appeared to be similar between both rats mLN Tn after IL-6 exposure, which is known to induce STAT3 phosphorylation (**Supplementary Figures S6E**, **S6F**).

Consistently, STAT1 deficiency was also present in thymic precursors of premorbid B27-rat Tn (**Supplementary Figure S7A**). Hence, *Stat1* mRNA expression was decreased in CD4^+^ single-positive (CD4SP) and/or CD4^+^CD8^+^ double-positive (DP) thymocytes of newborn (**Figure 4E**) and premorbid B27-rats (**Figure 4F**), whereas *Stat3* mRNA expression did not differ as compared to thymocytes from NTG rats (**Supplementary Figure S7B**). *Stat1* decrease was not due to the lower expression of upstream cytokines receptors (**Supplementary Figure S7C**). However, several STAT1-induced genes (*Usp18*, *Tbx21*, and *Ifit2*) were decreased in premorbid B27-rat CD4SP, whereas STAT3-induced genes (*Rorc*, *Batf*, and *Socs3*) did not differ from NTG rat cells (**Supplementary Figure S7C**). However, among the latter genes, *Il1r1* expression appeared already increased in premorbid B27-rat DP thymocytes (**Supplementary Figure S7D**). Noteworthy, concerning *Stat1* we observed comparable mRNA expression, between control B7- and NTG rats CD4SP (**Supplementary Figure S7E**).

Altogether, these data indicate that STAT1 deficiency is a very early event observed in newborn, preceding the onset of rat-SpA and already occurring in thymic precursors of Tn, most evidently in CD4SP subtype. Moreover, this deficiency seems to be secondarily associated with STAT3 increase upon rat-SpA development.

### STAT1 deficiency is characteristic of SpA patients Tn

**Figure 5:**
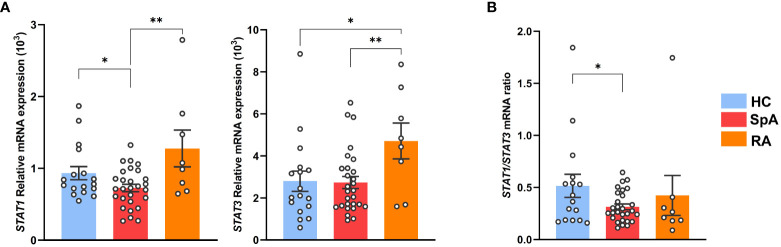
*STAT1* deficiency is characteristic of SpA patients Tn. Tn sorted from the PBMC of SpA patients (*n* = 28), RA patients (*n* = 8), and HC (*n* = 16). **(A)** *STAT1* (left) or *STAT3* (right) mRNA expression assessed by q-RT-PCR. **(B)** Ratio of *STAT1*/*STAT3* genes mRNA expression. Bar charts represent the mean ± SEM; each dot represents an individual patient. one-way analysis of variance showed significant difference between groups (*p* < 0.01). Groups comparisons were performed by Tukey’s test; *p < 0.05 and **p < 0.01.

We next evaluated *STAT1* expression in circulating Tn from axial SpA patients, of which 95% were HLA-B27^+^, in comparison with HC and RA patients (**Supplementary Table S1**, **Supplementary Figure S7A**). *STAT1* mRNA expression was decreased in Tn from SpA patients, as compared to both HC and RA patient cells, whereas *STAT3* appeared increased in RA patients as compared to both other groups (**Figure 5A**, **Supplementary Figure S9B**). Low *STAT1*/*STAT3* ratio appeared as a specific signature of SpA patients Tn, suggesting that STAT1/STAT3 transcription factors imbalance might be a critical event during patients Tn differentiation and thus in SpA development (**Figure 5B**).

### *Stat1* deficiency is associated with *Il1r1* upregulation, favoring a Th17 differentiation bias

**Figure 6:**
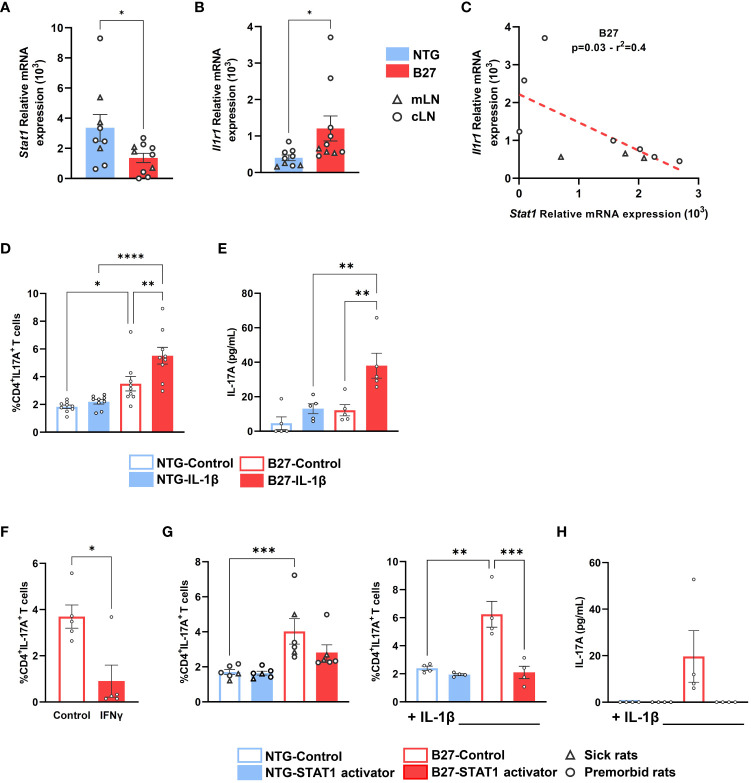
*Stat1* deficiency is associated with *Il1r1* upregulation, favoring a Th17 differentiation bias, that is inhibited by STAT1 signaling stimulation. Tn from **(A–C)** cLN and mLN or **(D–H)** mLN from **(A–E, G, H)** premorbid or sick **(F)** B27-rats and NTG age-matched littermates (*n* = 6–8). **(A**–**C)** *Stat1* and *Il1r1* mRNA expression assessed by q-RT-PCR. **(C)** Correlation between *Stat1* and *Il1r1* mRNA expression assessed by q-RT-PCR in B27-rat Tn. **(D–H)** Tn cultured in anti-CD3 coated wells in the presence of soluble anti-CD28 mAb, **(D, E, G, H)** with or without IL-1β, **(F)** with or without IFNγ, **(G, H)** with or without STAT1 transcriptional activator (2NP; 50 µM), for 3 days. **(D, F, G)** Intra-cellular expression of IL-17A was analyzed by flow cytometry, gated on live CD4^+^ T cells. **(E, H)** After 3 days, IL-17A was assessed by ELISA in supernatants. Bar charts represent the mean ± SEM; each dot represents an individual animal. **(D, E, G, H)** Two-way analysis of variance showed significant effect of genotype (*p* < 0.01) and treatment condition (*p* < 0.01) in all comparisons, without significant interaction. Groups comparisons were performed by **(A, B, F)** unpaired t-test or **(C)** linear regression or **(D, E, G, H)** Tukey’s test; *p < 0.05, **p < 0.01, ***p < 0.001, ****p < 0.0001.

To investigate whether decrease of *Stat1* expression was related to the tissular inflammatory environment, we evaluated its expression in Tn from cLN, that is, draining a tissue irrelevant for rat-SpA. Interestingly, *Stat1* decrease was observed similarly in Tn from premorbid B27-rat cLN and mLN, the latter draining the gut, that is, a rat-SpA target tissue, as compared to NTG rat cells (**Figure 6A**). This suggested that this signature might be ubiquitous in B27-rat and favor proinflammatory differentiation bias of Tn in a tissue-dependent way, which could rely on locally secreted cytokines. IL-1β is a major cytokine secreted by inflammatory tissues (37). Interestingly, the upregulation of IL-1β receptor gene, *Il1r1*, appeared as an early event in mLN B27-rat Tn (**Figures 2B, D, E**, **3C**, **Supplementary Figure S7D**). Thus, we examined the putative link between *Stat1* decrease, IL-1 pathway and Th17 bias. We confirmed that *Il1r1* was increased in cLN as well as in mLN B27-rat Tn and observed that *Stat1* was negatively correlated with *Il1r1* expression, suggesting that they may cross-regulate each other (**Figure 6B, C**). Hence, addition of rrIL-1β during Tn activation exacerbated IL-17A production in premorbid B27 but not NTG rat mLN Tn (**Figures 6D, E**). Thus, the sensitivity of mLN B27-rat Tn to IL-1β reinforced Th17-biased differentiation, possibly related to *Il1r1* upregulation and *Stat1* deficiency.

### STAT1 signaling in B27-rat Tn inhibits IL-17A expression

We finally examined the functional consequences of STAT1 deficiency on Tn differentiation. We first demonstrated that Th1 differentiation of B27-rat Tn was unaffected (data not shown), despite the previously described decreased basal level of STAT1. Interestingly, the addition of IFNγ, which signals through STAT1, induced a decrease of IL-17A production (**Figure 6F**). The addition of 2NP, a selective STAT1 transcriptional activator, during Tn activation specifically reversed the increased IL-17A expression and production in premorbid and sick B27-rat T cells (**Figure 6F**), even more strikingly in the presence of IL-1β (**Figures 6G, H**). We thus confirmed that activation of STAT1 signaling could reverse the Th17 bias imprinted in B27-rat Tn.

## Discussion

Rat-SpA that develops spontaneously in the 33-3 line of B27-rats appears dependent on CD4^+^ T cells and is associated with abnormal expansion of proinflammatory Th17 cells producing TNFα and IL-17A, favored by abnormal signals delivered by APC (3, 5, 38). Consistently, B27-rat DCs exhibit altered functions participating to rat-SpA susceptibility and shared with SpA patients, including reverse-IFN signature and decreased IL-27 and IL-10 expression that could contribute to Th17 expansion (7, 8, 39).

Here, we highlighted the critical role of a precocious DCs/Tn abnormal cross-talk, as premorbid B27-rats cDC2 induced heightened activation and Th17-like profile in cocultured Tn. Moreover, we revealed that B27-rat Tn were prone to develop a proinflammatory profile before rat-SpA onset. The analysis of the B27-rat Tn transcriptome showed two opposite signatures: defective Th1/IFN and heightened Th17 patterns, notably involving key transcription factors of both pathways, that is, STAT1 and STAT3, respectively. This appears particularly compelling, since transcription factor from both pathways cross-regulate each other (29, 40–43). This imbalanced transcriptomic signature between both pathways was present in premorbid rats and exacerbated in sick B27-rats, providing further confirmation of its relevance to the rat-SpA disease process.

Epigenomic study of histone marks associated with active promoters, enhancers, and SE regions consistently revealed that B27-rat Tn were imprinted at the chromatin level to differentiate preferentially into Th17 cells. Corroborating this observation, IRF4 and BATF—pioneer transcription factors favoring STAT3 binding and transcriptional activity—were predicted to bind to the SE specific of B27-rat Tn (44). In mirror, a decreased binding of STAT1 was predicted on the SE of B27-rat Tn, which also probably reinforced Th17-skewed differentiation of B27-rat Tn (43).

We next examined the earliest stage of CD4^+^ T cell development in B27-rat and found that several of the IFN pathway-related genes (*Stat1*, *Usp18*, *Tbx21*, and *Ifit2*) were already downregulated in the developing thymocytes from premorbid and/or even newborn B27-rats, in particular in the CD4SP. This could suggest a specific role of early biased signals delivered by DC during thymic selection, inducing epigenetic imprinting (45). In contrast, this was not the case for the Th17 pathway–related genes, to the exception of *Il1r1*. Thus, it suggests that the primary alteration in B27-rat Tn most likely resides in the reverse-IFN signature and particularly in decreased *Stat1* expression. This could promote a secondary increase in STAT3/Th17 response which is a hallmark of established rat-SpA (5). This interpretation is consistent with the decrease of STAT1 protein and phosphorylation in premorbid B27-rat Tn, whereas increased STAT3 was only detected in sick B27-rat Tn. It is also supported by the negative regulation of IL-17 production induced *in vitro* by addition of IFNγ or a transcriptional activator of STAT1 during stimulation of B27-rat Tn. Noteworthy, Th17 expansion was also inhibited by addition of IL-27, a pro-Th1 cytokine, in B27-rat CD4^+^ T cultures, restoring the balance between STAT1 and STAT3 activation and *in vivo*, allowing to prevent rat-SpA development (36).

Concerning *Il1r1* upregulation detected in B27-rat thymocytes, it was possibly a consequence of *Stat1* downregulation as suggested by the negative correlation observed between both genes expression in our model, and the already known mutual antagonism between IFN and IL-1 signaling pathways (46). Increased expression of this cytokine receptor gene could contribute to enhance the differentiation of proinflammatory Th17 cells in sites where IL-1 is produced, notably in the gut upon exposure to microbiota, which is a required trigger of rat-SpA in the 33-3 line (47–49).

As in the B27-rat, SpA patients display a Th17 bias that appears to be of critically importance, as confirmed by the efficacy of anti–IL-17 antibodies treatment in a significant number of patients (50, 51). Thus, our observation of a similarly decreased *STAT1* expression and *STAT1*/*STAT3* imbalance in Tn from HLA-B27^+^ SpA patients supports the relevance of this observation to human SpA development. Indeed, STAT1 deficiency could favor a Th17 bias in patients but other factors might contribute to the complex pathophysiology of SpA. Consistently, decreased T cell IFNγ response as well as an expansion of EBV- and CMV-specific CD8^+^ T-cell clonotypes have previously been reported in SpA patients that could indicate a defective control of endogenous viruses (52, 53), as a result of altered *STAT1* expression.

In addition to Tn, it is striking that other types of immune cells from B27-rat, that is, cDC2 (8) and cDC1 (Fert I, Araujo LM, Breban M, unpublished observation) as well as monocytes-derived macrophages from SpA patients (8, 54) share a similar reverse-IFN signature, including the downregulation of *Stat1*/*STAT1*, *Isg15*, *Irf7*, *Oasl*, and *Usp18*. Given that reverse-IFN signature is observed very precociously in thymocytes and similarly in myeloid lineages from B27-rats and SpA patients, it probably occurs as a cellular consequence of HLA-B27 expression. The molecular events linking HLA-B27 to this signature remains to be determined, but we have previously evidenced non-canonical behaviors of the HLA-B27 molecule, including intracellular trafficking alteration (55) and interaction with BMP/TGFβ receptor family signaling-pathways (56) that could potentially result in reshaping immune cells function/differentiation. In turn, STAT1 deficiency could favor Th17 differentiation either directly by altering the cellular response to stimulation (40), and/or indirectly by affecting the control of gut microbiota, which is consistent with the gut dysbiosis that is present both in B27-rat and SpA patients (57, 58).

The causal role of STAT1/STAT3 transcription factors imbalance in Th17-biased differentiation of B27-rat Tn is supported by the anti-IL17 effect of transcriptional activator of STAT1 during Tn stimulation, and by our recently published work showing that IL-27 presents anti-inflammatory effects during rat-SpA via the activation of STAT1 and the suppression of Th17 responses (36). Altogether, these data offer the innovative perspective that reshaping the differentiation of Tn early on during the disease process, could prevent an accumulation of pathogenic Th17 population during the course of SpA.

In summary, B27-rat Tn harbor a STAT1 deficiency preceding disease onset, secondarily associated with a persistent Th17 bias, imprinted at the epigenomic level, and probably related to HLA-B27 expression. This early molecular phenomenon may contribute to the persistent proinflammatory skew of CD4^+^ T cells in SpA patients and provide insights for a better understanding and treatment of SpA.

## Data availability statement

The original contributions presented in the study are included in the article/**Supplementary Material**, further inquiries can be directed to the corresponding author. The data presented in the study are deposited in the GEO repository, accession number GSE245067. 

## Ethics statement

The studies involving humans were approved by Local ethical committee (Comité de Protection des Personnes Ile-de-France XI, AOR10006-NI09031). The studies were conducted in accordance with the local legislation and institutional requirements. The participants provided their written informed consent to participate in this study. The animal study was approved by Institutional Animal Experimentation Ethical Committee from the Faculty of Health Simone Veil (APAFIS-8910). The study was conducted in accordance with the local legislation and institutional requirements.

## Author contributions

Conceptualization: BC, FCr, LMA, MB; Methodology: BC, FC, ML, GS, RS-N, HM, FCo, MF, AH, H-JG, SG, LMA, MB: Investigation: BC, FC, ML, GS, RS-N, HM, FCo, MF, SG, LMA, MB; Visualization: BC, FCr, ML, GS, RS-N, HM, FCo, MF, SG, LMA, MB; Funding acquisition: BC, FCr, LMA, MB; Project administration: FCr, LMA, MB; Supervision: FCr, LMA, MB; Writing – original draft: BC; Writing – review & editing: BC, FCr, LMA, MB. All authors agree to be accountable for the content of the work. All authors contributed to the article and approved the submitted version.
