# Supplementary material for: STAT1 deficiency underlies a proinflammatory imprint of naive CD4+ T cells in spondyloarthritis
Source: Front Immunol. 2023 Oct 18;14:1227281. doi: 10.3389/fimmu.2023.1227281 (PMC10619905; doi:10.3389/fimmu.2023.1227281)
Supplement: Supplementary file 1 [file DataSheet_1.docx]

Supplementary Material

STAT1 deficiency underlies a proinflammatory imprint
of naive CD4^+^ T cells in spondyloarthritis

Bilade Cherqaoui^1,2^, Frédéric Crémazy^1,2^, Marc Lauraine^1,2^, Ghazal Shammas^1,2^, Roula Said Nahal^3^ MD, Hendrick Mambu Mambueni^1,2,4^, Félicie Costantino^1,2,3^, Marine Fourmont^1,2^, Audrey Hulot^1,2^, Henri-Jean Garchon^1,2,4^, Simon Glatigny^1,2^, Luiza M. Araujo^1,2^†, Maxime Breban^1,2,3^†*

^1^Infection & Inflammation, UMR 1173, Inserm, UVSQ/Université Paris Saclay, Montigny-le-Bretonneux, France.

^2^ Laboratoire d’Excellence Inflamex, Université Paris-Centre, Paris, France.

^3^Rheumatology Division, Ambroise Paré Hospital, AP-HP, Boulogne-Billancourt, France.

^4^Genomic platform of Faculty of Health Simone Veil, UVSQ/Université Paris Saclay, Montigny-le-Bretonneux, France.

***Corresponding** **author**: 2, avenue de la Source de la Bièvre – 78180 Montigny-le-Bretonneux (France); maxime.breban@aphp.fr, +33 149 095 672, fax : +33 149 094 555

† These authors share last authorship

**Supplementary Figure 1.**

**
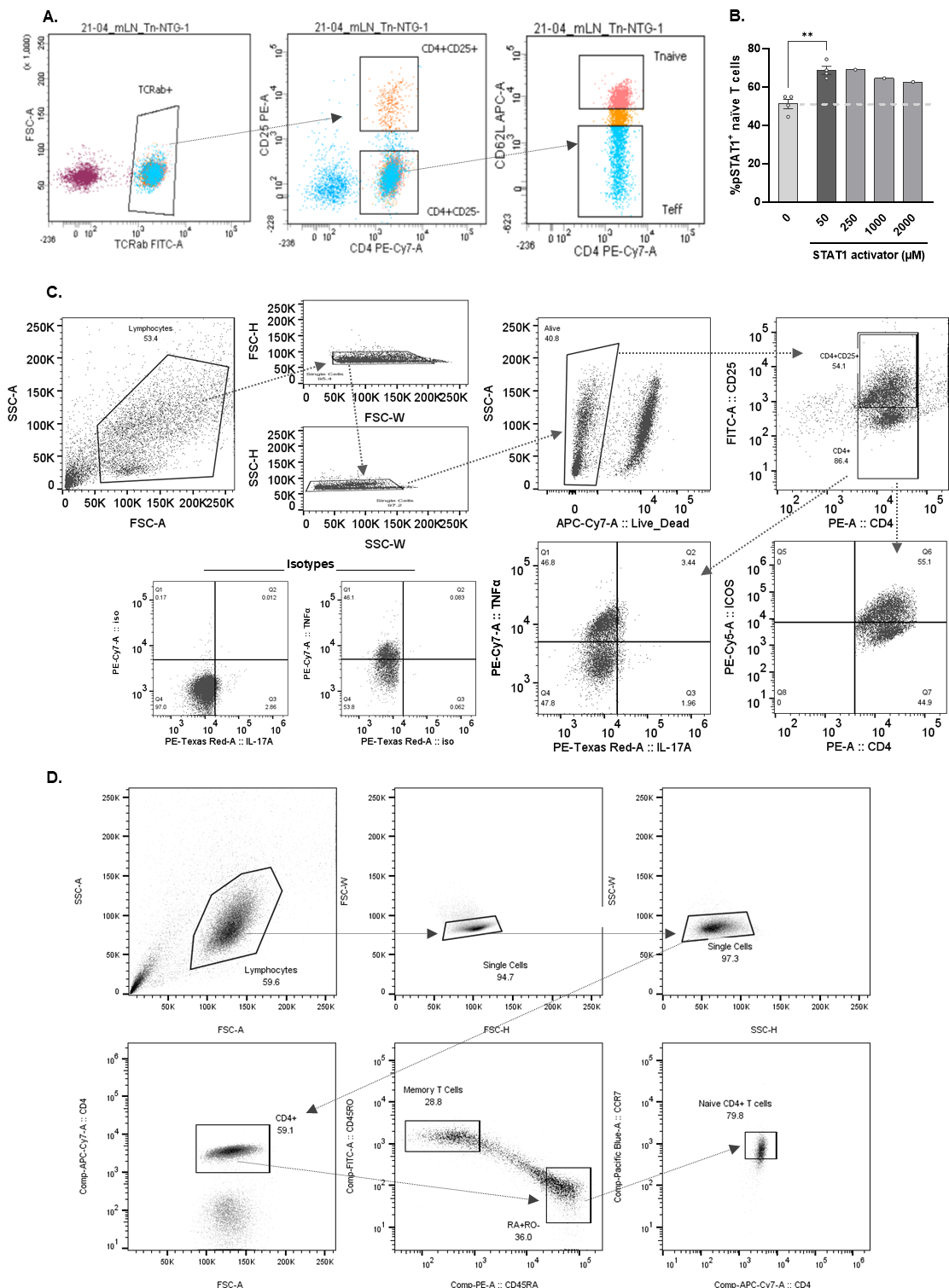
**

(**A**) Sorting strategy to isolate Tn from sick rat mLN is shown; similar sorting strategy was used for premorbid rats Tn. (**B**) Single cell suspension was obtained from mLN of 3-5 mo-old NTG rats. Phosphorylated STAT1 MFI was assessed by flow cytometry in Tn after IL-27 stimulation for 10 minutes, in the presence or not of STAT1 transcriptional activator 2NP at various concentrations. Bar charts represent the mean ± SEM of 4 independent experiments. *p*-value was calculated by paired t-test; ** p<0.01. (**C**) After 3 d of Tn culture with or without cDC2, cell surface or intra-cellular expression of CD25, ICOS, TNFα and IL-17A was analyzed by flow cytometry, gated on live CD4^+^ T. An example of gating strategy is shown, including isotype controls for IL17A and TNFα. (**D**) Human Tn were sorted from the PBMC of axial SpA patients and controls, as shown in sorting strategy.

**Supplementary Figure 2.**

**
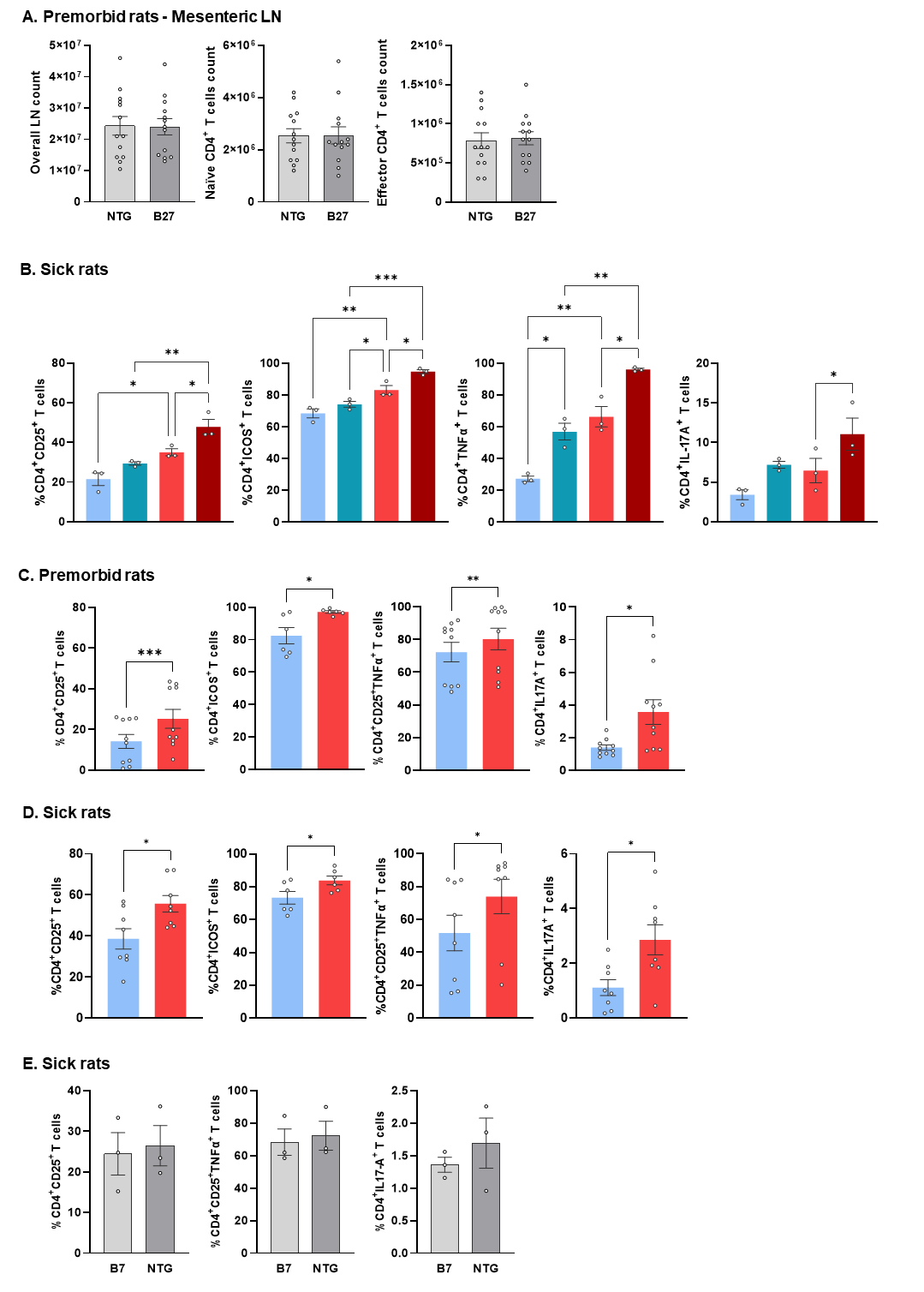
**

Cells were sorted from (**A,C**) premorbid B27-rats (n = 10-12) or (**B,D**) sick B27-rats (n = 4-8) or **(E)** adult B7-rats and age-matched NTG littermates. (**A**) The absolute number of mLN cells was counted *ex vivo*; the count of Tn and effector CD4^+^ T cells was estimated after *ex vivo* cell sorting. (**B**) Cocultures of splenic cDC2 with mLN Tn, or (**C,D**) monoculture of stimulated mLN Tn, were performed. (**B-E**) After 3 d, CD4^+^ T cells were analyzed by flow cytometry for cell-surface expression of CD25 and ICOS, and intra-cellular expression of TNFα and IL-17A. Data are gated on live CD4^+^ T cells. Bar charts represent the mean ± SEM. Each dot represents an independent coculture. *p*-values were calculated by (**A,C,D**) unpaired t-test or (**B**) 2-way Anova; * *p*<0.05, ** *p*<0.01, *** *p*<0.001.

**Supplementary Figure 3.**

**
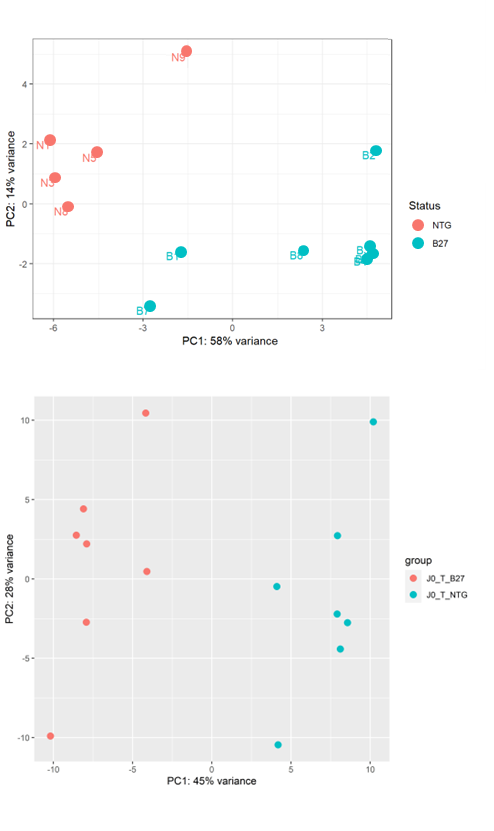
**

RNA-seq was performed on mLN Tn from 3 wk-old premorbid B27-rats and NTG littermates (upper panel), and 3 mo-old sick B27-rats and NTG littermates (lower panel). Principal component analysis plot, after batch-correction, showing a clearcut segregation between B27- and NTG rats mLN Tn.

**Supplementary Figure 4.**

**
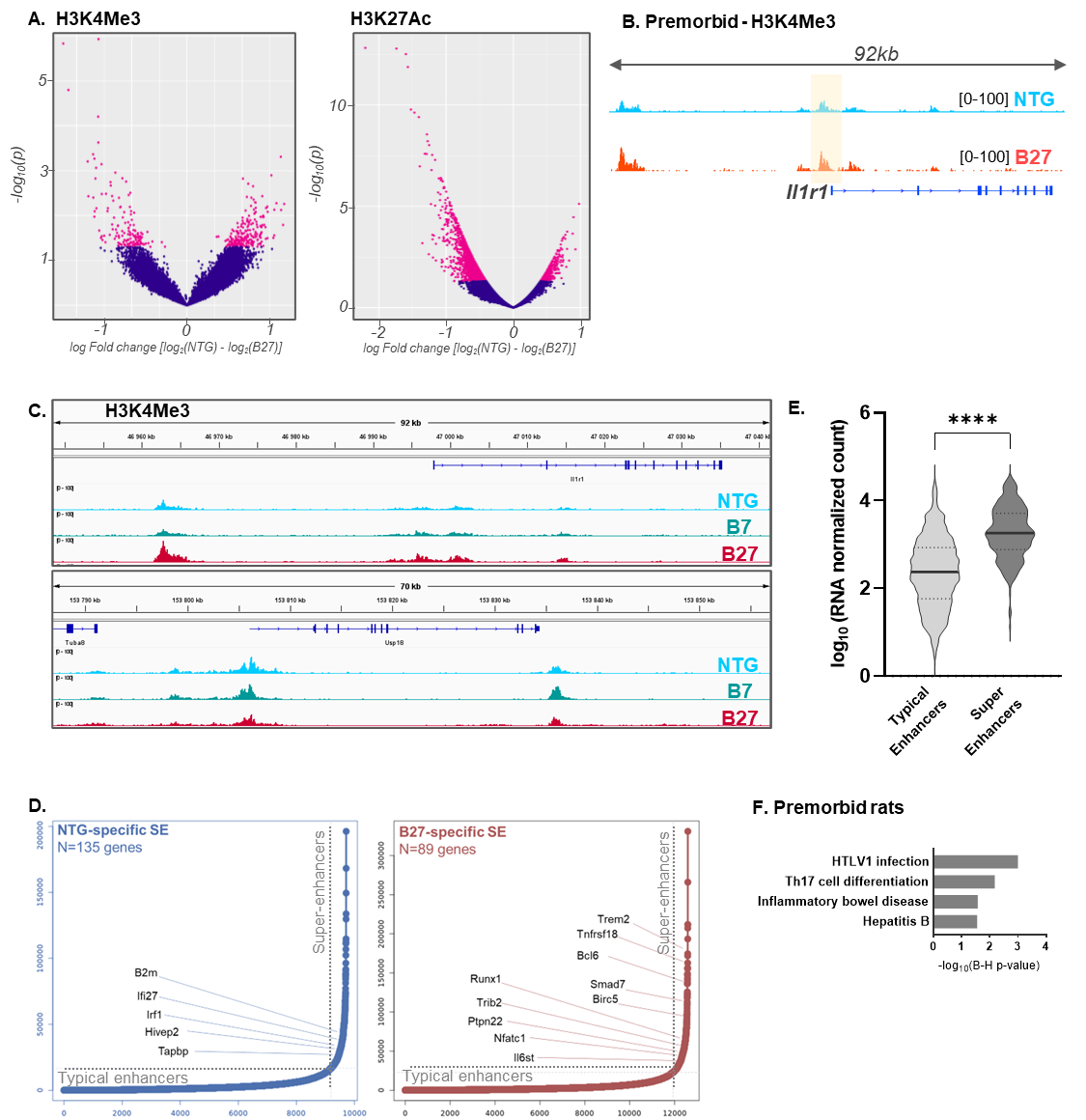
**

ChIP-seq was performed with Abs specific for H3K4Me3 and H3K27Ac HM on mLN Tn sorted from (**A,C-E**) 3 mo-old sick B27-rats (n = 3) or from (**B,F**) 3 wk-old premorbid B27-rats (n = 2). Age-matched (**A,B,D-F**) NTG littermates and (**C**) 3 mo-old B7-rats were used as controls. (**A**) Volcano plots displaying log fold-change versus -log_10_ adjusted *p-*value for each H3K4Me3 peak (left panel) and H3K27Ac peak (right panel) enriched in B27-rats, as compared to NTG littermates Tn. Peaks considered as significantly differentially enriched (adjusted *p* < 0.05) are labeled in pink. (**B**) Integrated genome viewer snapshots of representative gene involved in Th17 differentiation (*Il1r1*) comparing premorbid B27-rat and NTG littermate conditions. Graphed dataset from 1 pair of B27/NTG rats, representative of 2. (**C**) Integrated genome viewer snapshots of genes involved in Th17 differentiation (*Il1r1*, upper panel) and IFN (*Usp18*, lower panel) pathways. Graphed dataset from 1 trio of B27/NTG/B7 rats, representative of 2. (**D**) Graph showing SE of B27-rats (left) as compared to those of NTG littermates (right) Tn. SE-related genes involved in IFN (blue) and Th17 (red) pathways are labeled. (**E**) Violin plot showing the mRNA normalized count extracted from RNA-seq data (expressed in log_10_) of genes located at proximity of typical enhancers as compared to those at proximity of SE. Median (thick line) and quartile (dotted line). *p*-value was calculated by unpaired t-test; **** *p*<0.0001. (**F**) The graph shows the 4 KEGG pathways enriched for TF predicted to bind on B27-SE of Tn. Adjusted *p* values expressed in -log_10_.

**Supplementary Figure 5.**


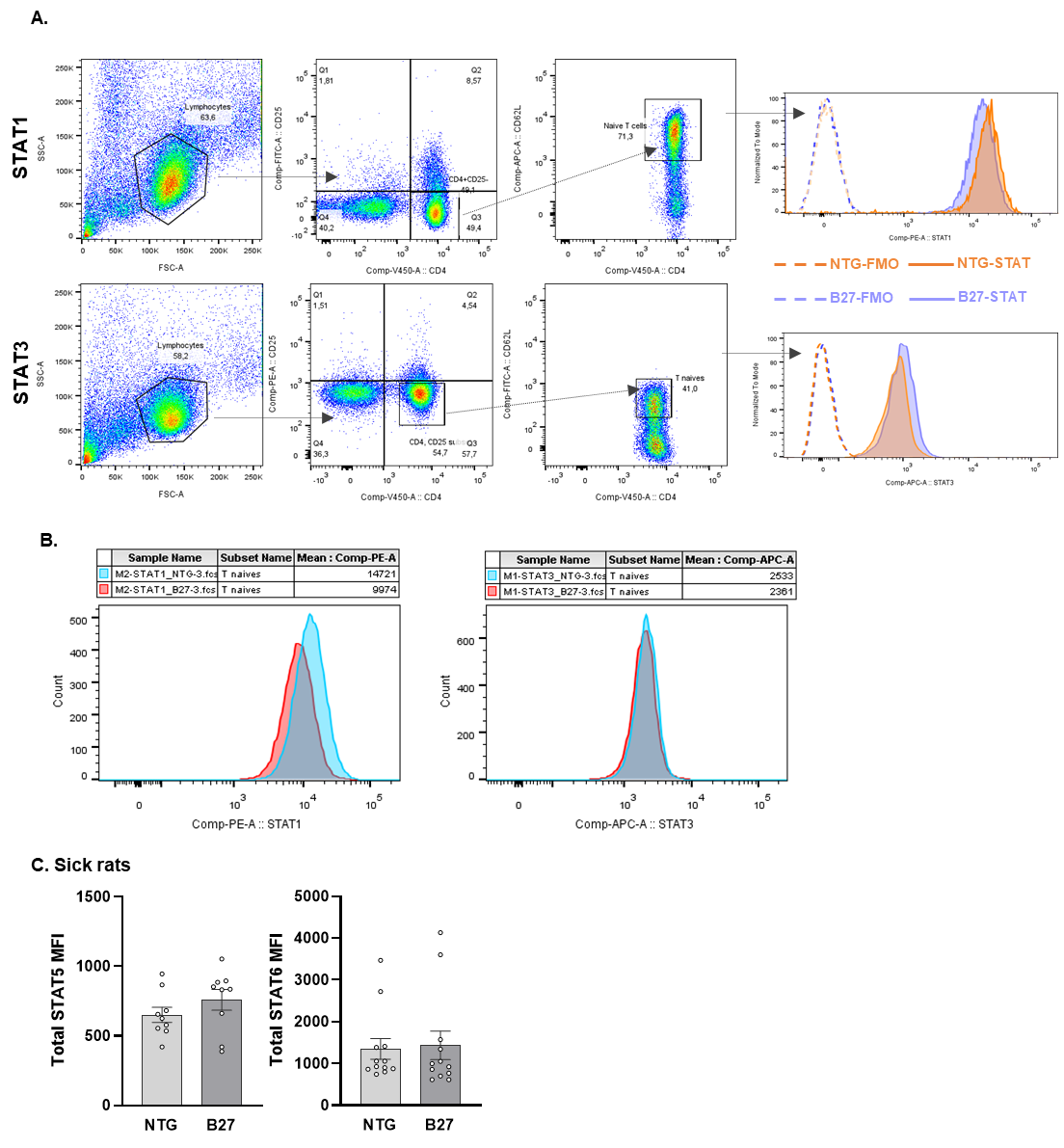


Single cell suspension was obtained from mLN. Total STAT MFI was assessed by flow cytometry in Tn. (**A**) Gating strategy of Tn in adult rat (left) and FMO control for STAT1 and STAT3 staining (right). (**B**) Overlapping representative histograms of STAT1 and STAT3 MFI in NTG vs B27 gated Tn in adult rat. (**C**) Total STAT5 (left panel) and STAT6 (right panel) in sick B27-rats and NTG littermates, bar charts represent the mean ± SEM of 8 to 12 rats per group. *p*-values were calculated by paired t-test.

**Supplementary Figure 6.**


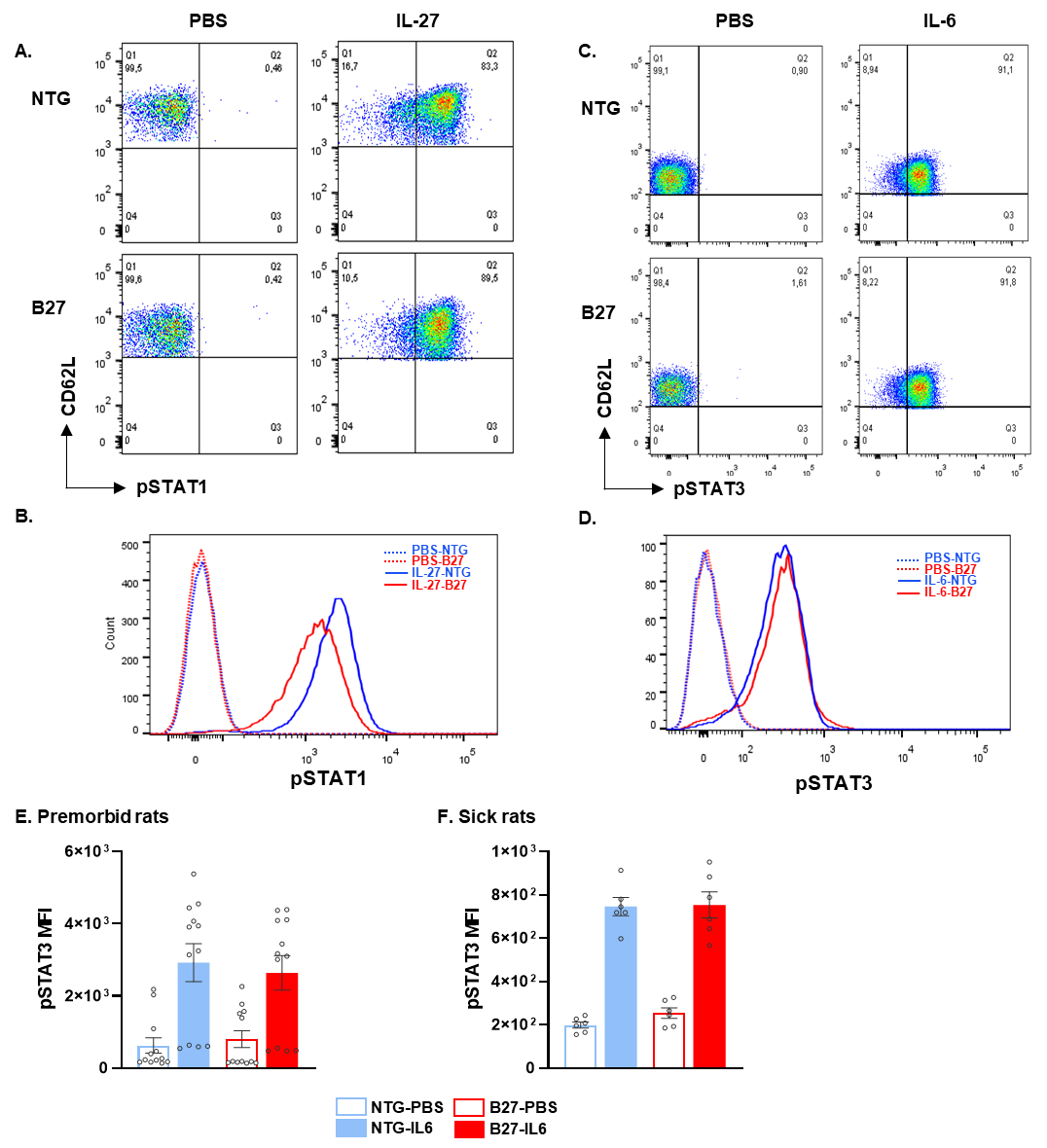


Single cell suspension was obtained from mLN. Cells were stimulated with control (PBS) or IL-27 (for STAT1 measurement) or IL-6 (for STAT3 measurement) for 10 minutes. pSTAT MFI was assessed by flow cytometry in Tn. **(A,C**) Gating strategy of pSTAT1^+^ or pSTAT3^+^ Tn. (**B,D**) Overlapping representative histogram of pSTAT1 and pSTAT3 MFI level expression in NTG vs sick B27-rat gated Tn with or without stimulation. (**E,F**) Phosphorylated STAT3 MFI in (**E**) premorbid B27-rats or (**F**) sick B27-rats and NTG littermates. Bar charts represent the mean ± SEM of 6 to 12 rats per group.

**Supplementary Figure 7.**

**
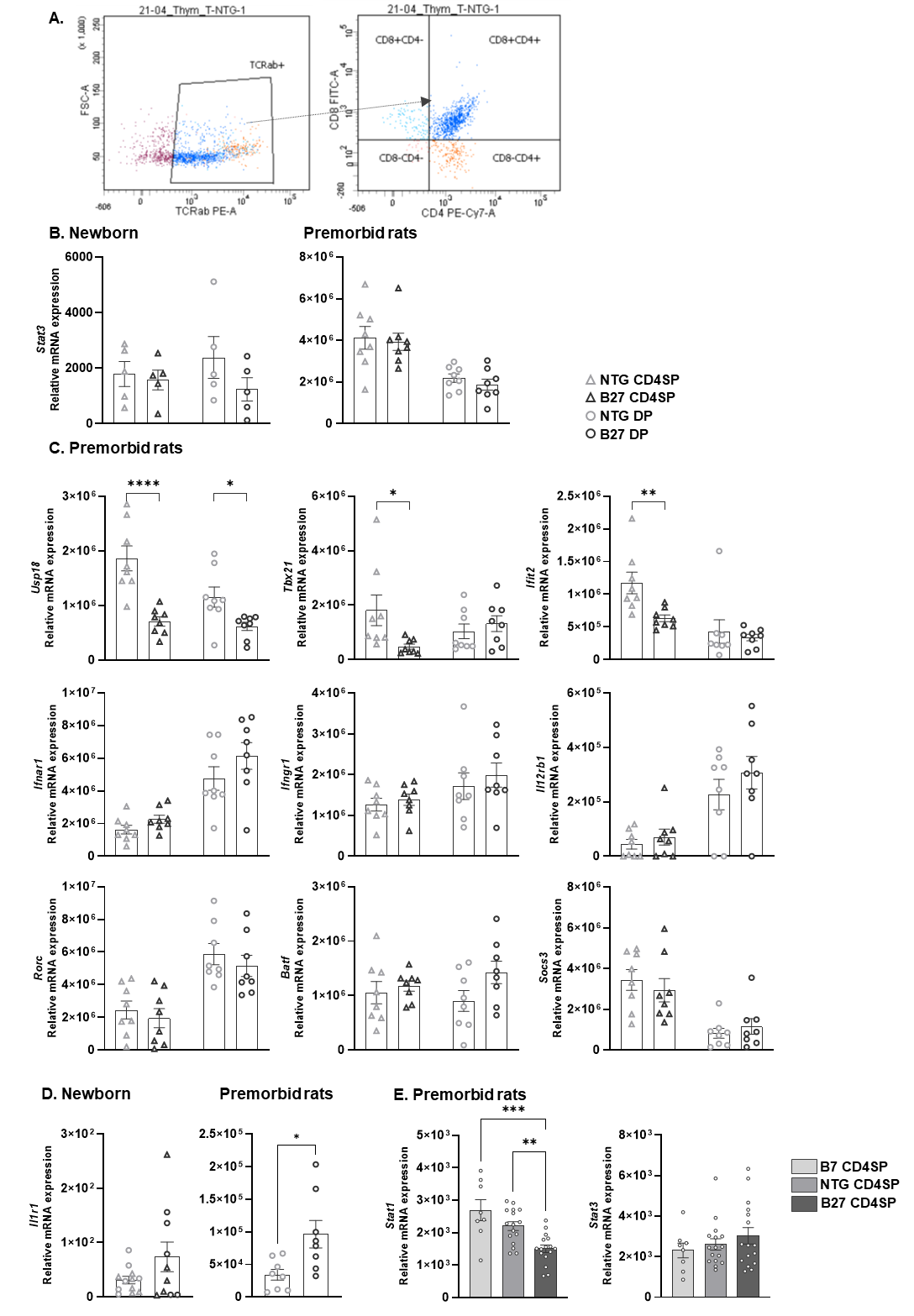
**

(**A**) Sorting strategy for thymic CD4^+^ single-positive (CD4SP) and/or CD4^+^CD8^+^ double-positive (DP) thymocytes. IFN/Th1 and Th17-related genes mRNA expression was assessed by q-RT-PCR in (**B-E**) premorbid, or (**B,D**) new-born B27-rats. Age-matched (**B-E**) NTG littermates and (**E**) B7-rats were used as controls. Bar charts represent the mean ± SEM of 5 to 8 rats per condition. *p*-values were calculated by unpaired t-test; * p<0.05, ** p<0.01, *** p<0.001, **** p<0.0001.
